# Morphological and Molecular Characterization of Selected Chilean Runner Bean (*Phaseolus coccineus* L.) Genotypes Shows Moderate Agronomic and Genetic Variability

**DOI:** 10.3390/plants10081688

**Published:** 2021-08-17

**Authors:** Osvin Arriagada, Andrés R. Schwember, María Jesús Greve, Milan O. Urban, Ricardo A. Cabeza, Basilio Carrasco

**Affiliations:** 1 Departamento de Ciencias Vegetales, Facultad de Agronomía e Ingeniería Forestal, Pontificia Universidad Católica de Chile, Santiago 7820436, Chile; arriagada.lagos.o@gmail.com (O.A.); aschwember@uc.cl (A.R.S.); mjgreve@uc.cl (M.J.G.); 2 Bean Physiology Team, International Center for Tropical Agriculture (CIAT), Cali 763537, Colombia; m.urban@cgiar.org; 3 Departamento de Producción Agrícola, Facultad de Ciencias Agrarias, Universidad de Talca, Talca 3460000, Chile; rcabeza@utalca.cl; 4 Scientific Director at Centro de Estudios en Alimentos Procesados (CEAP), Av. Lircay s/n, Talca 3460000, Chile

**Keywords:** runner bean, morphology, ISSR markers, genetic diversity, *Phaseolus* spp.

## Abstract

The runner bean is the third most economically important *Phaseolus* species, which is cultivated on small-scale agriculture for the production of immature pods or to obtain dry seeds. However, despite the economic importance and agronomic potential of this species, the runner bean has been little studied from the genetic standpoint. Therefore, the main objective of this study was to characterize ten selected lines of runner bean obtained from Central (Santiago) and Southern (Valdivia and Villarica) Chile based on morphological and agronomic traits. In addition, the genetic variability of these lines was determined using 12 Inter-Simple Sequence Repeat (ISSR) markers to evaluate the potential of this germplasm for breeding and commercial purposes. As a result, the lines from Central Chile were characterized, and had a higher number of pods per plant compared to the Southern lines, although the size and weight of their seeds were lower. Moreover, a low level of genetic diversity (*He* = 0.251) was encountered in this population. Finally, this is one of the first studies that generate relevant and novel information on the morphological, agronomic and genetic characterization of the *P. coccineus* germplasm present in Chile.

## 1. Introduction

The genus *Phaseolus*, belonging to the *Fabaceae* family, includes over 70 species, of which five were domesticated in Mesoamerica and have distinct geographical distributions, life histories and reproductive systems [1]. The (scarlet) runner bean (*P. coccineus* L.) is the third most economically important *Phaseolus* species worldwide, after the common bean (*P. vulgaris* L.) and lima bean (*P. lunatus* L.) [2]. It is a climbing long-day perennial legume species that is often cultivated as a small-scale annual crop for edible immature pods production or to obtain dry seeds [3]. In addition, its flowers (generally red flowers) are used as food, or to decorate salads, soups, appetizers, desserts and drinks [4]. Based on the color of the flowers, taxonomists described three botanical varieties of runner beans: *P. coccineus* var. *albiflorus* with white flowers, *P. coccineus* var. *coccineus* with red flowers and *P. coccineus* var. *bicolor* with both colors (white and red) of flowers [5].

Wild populations of runner bean are found from Northern Mexico to Panama, in cool and humid uplands, at altitudes from 1000 to 3000 m.a.s.l. [2,6], due to low temperature requirements for seed germination and plant growth [7]. However, the runner beans are currently grown commercially in Europe (the United Kingdom, Italy and Spain [5,7,8]), Mesoamerica (Mexico, Guatemala, Honduras and Costa Rica [7,9]) and to a lesser extent in Africa (Kenya [10,11]) and South America (Argentina and Chile [3,12]).

Runner bean is highly allogamous, and is a source of variability for several agronomic and disease resistance traits for the improvement of the common bean [1,8]. Some agronomic traits of interest are lodging resistance due to thick stem bases, cold tolerance, the presence of tuberous roots, allowing a perennial cycle and likely drought resistance, and a potentially high number of pods per inflorescence [3,8]. Furthermore, alleles associated with resistance to pathogens such as *Sclerotinia sclerotiorum* [13] and *Xanthomonas campestris* [14] have been introgressed into common bean by crossing with *P. coccineus*. However, despite the economic importance and agronomic potential of runner bean, the lack of molecular characterization in germplasm collections restricts its overall utilization, especially as an interspecific hybridization donor species in *Phaseolus* breeding programs [2,15].

Determining the level of genetic diversity and relationships among genotypes are fundamental steps in any genetic improvement program. Some studies have confirmed that *P. coccineus* presents a wide variability in morpho-agronomic traits, and low to moderate levels of genetic diversity in wild and cultivated populations, being little affected by the domestication process [1,2,16,17,18,19]. In this sense, demographic expansion and the introgression of alleles from wild relatives are some of the factors that have increased and/or maintained the genetic diversity in cultivated populations of *P. coccineus* [20]. However, few studies have focused on the improvements of *P. coccineus* itself, and therefore there are few breeding programs focused on this species in the world, mainly in Europe [11].

In Chile, there are small home-made vegetable gardens with runner bean grown in the Southern area of the country [3]. According to Tay [21], there is an interesting genetic diversity in the Chilean runner bean germplasms, with large-seeded genotypes with different colors such as white, black and marbled. However, not much attention has been paid to this species from a genetic standpoint, despite its excellent culinary quality and the morpho-agronomic variability that exists in the country [12,21,22]. In this context, the objective of the present work was to characterize ten Chilean lines of runner bean from different origins (Central and Southern Chile) using selected morphological and agronomical traits. In addition, these lines were studied to determine their genetic variability through ISSR markers, and thus evaluate the potential of this germplasm for breeding and commercial purposes.

## 2. Results and Discussion

### 2.1. Qualitative Morphological Characterization

**Figure 1:**
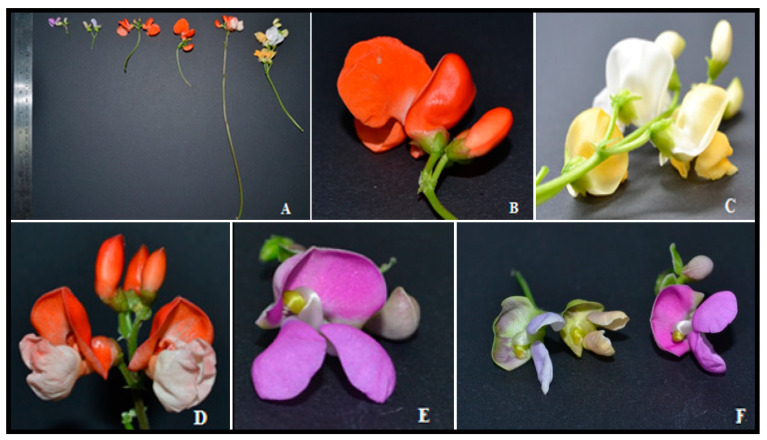
Morphology and colors present in the flowers of the ten Chilean lines of *P. coccineus* evaluated. (**A**) The different colors, petiole and flower size. (**B**) Orange flower morphology. (**C**) White flowers and the color change as the flower fades. (**D**) Color distribution in orange and white flowers. (**E**) Purple flower, and (**F**) similarity between the flowers of *P. lunatus* (left) and line 3 (right).

Germination type is often indicative of evolutionary changes among clades [23], and one of the most relevant morphological traits that differentiates species [24]. According to Delgado-Salinas [6], *P. coccineus* subsp. *coccineus* and the wild subspecies have hypogeal germination, while *P. coccineus* subsp. *darwinianus* has epigeal germination. In this study, six lines (1, 8, 19, 20, 27, and 28) showed hypogeal germination, while four lines (3, 4, 6, and 7) exhibited the epigeal type of germination, which is typical of *P. vulgaris* [4] (Table 1). The hypogeal germination is an adaptation of the species, and it facilitates avoiding cold environments at an early stage of growth [25]. Three botanical varieties of *P. coccineus* have been described according to the color of the flowers [5]. Among the 10 lines of *P. coccineus* evaluated, six lines (1, 4, 6, 7, 8 and 20) had white flowers, most of them coming from INIA (Chile’s National Agricultural Research Institute)-La Platina, Santiago, Chile. Orange flowers were only observed in the line 19 (Austral University of Chile, UACH, Valdivia). The lines from Pontificia Universidad Católica de Chile (PUC), Villarica, Chile (lines 27 and 28) displayed bicolored flowers. Line 3 presented purple/violet flowers, which were very similar to flowers of the *P. lunatus* species (Figure 1), indicating a possible hybridization crossing between species. Lines of *P. coccineus* with purple flowers have been described in previous studies using populations from Mexico [2,26] and Italy [27]. Moreover, *P. coccineus* var. *coccineus* with purple/violet flowers are associated with resistance to low temperatures [5].

One of the main traits selected during the domestication of legumes is the growth habit, in which plants with few branches, shorter internodes, fewer nodes, reduced twining and a determinate stem ending are desirable [28]. In fact, most domesticated beans have a determinate growth habit, which allows a decrease in plant biomass and optimized allocation between vegetative and reproductive growth [28]. Conversely, wild common beans are all indeterminate, as well as a sizable fraction of domesticated beans [28]. The runner bean is characterized by an indeterminate type of growth [4], which coincides with our results where all the lines exhibited an indeterminate growth type, excepting line 3, which 50% of the plants showed a determined growth habit (Table 1). Similar results were reported by Sinkovic et al. [15], who evaluated a population of 142 accessions of runner bean from South Eastern Europe, and almost all of accessions (excepting three) exhibited an indeterminate plant growth. In addition, Sicard et al. [27] evaluated nine local varieties of *P. coccineus* from Eastern Italy, and all the varieties showed an indeterminate plant growth. From a molecular point of view, in common bean, the determinate/indeterminate growth habit is regulated by the *PvTFL1y* gene, which is an ortholog of Arabidopsis *Terminal Flowering 1* (*TFL1*) [28,29]. Mutations in the *PvTFL1y* gene generate plants with a determined growth habit, which could explain the phenomenon that occurred in line 3. The number and the distribution of branches depend on agronomic management practices, environmental conditions and genotype used [30]. 70% of the lines evaluated had branches throughout the entire plant (evenly distributed; lines 1, 4, 6, 7, 8, 19 and 20). The lines 27 and 28 presented branches evenly distributed and upper (branches present above the fifth node of the plant) in equal frequency, whereas line 3 presented lower branches (branches present under the fifth node of the plant) and evenly distributed. These tiny changes in the branching architecture can be associated to different photothermal requirements of these lines since those with upper branching (lines 27 and 28) are from Southern Chile (PUC-Villarica), whereas the one with lower branching (line 3) is from Central Chile (INIA-La Platina). However, there is no previous background on the literature of *P. coccineus* that refers specifically to this particular trait.

Given that runner bean is usually grown for immature green pod production or to obtain dry seeds [1,3], the color of pods in these species is a very important trait. The color of pods ranged from cream to beige in all lines. However, lines 3 and 4 presented purple and brown spots, respectively (Table 2; Figure S1). In relation to the seed-related traits, most of the lines evaluated (70%) presented kidney-shaped seeds. Moreover, all the lines exhibited hilum of light coloration, which varied between white and beige.

**Figure 2:**
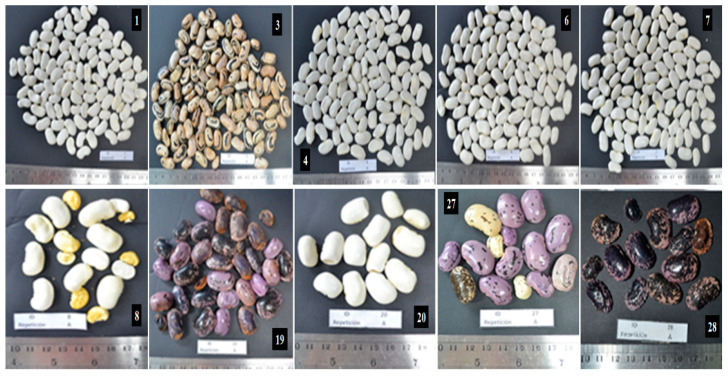
Shape and color of the seeds of each of the *P. coccineus* lines evaluated. Lines 1, 3, 4, 6, 7 and 8 come from the Central zone of Chile (INIA-La Platina), and lines 19, 20, 27 and 28 come from the Southern zone of Chile (UACH and PUC-Villarica).

The appearance of the seed, in particular the color, is the most critical aspect that affects consumer preferences. The light cream-colored seeds with brown spots is the preferred testa color distribution [31]. Six lines (1, 4, 6, 7, 8 and 20) presented seeds with white color, line 3 had beige-colored seeds with black spots, the lines 19, 27 and 28 showed purple seeds with black spots (Table 2; Figure 2). This color pattern was also observed in the study carried out by Rodríguez et al. [5] using three *P. coccineus* varieties, in which the var. *albiflorus* presented white seeds, the var. *bicolor* had beige seed and brown spots, and the var. *coccineus* exhibited purple seed and black spots. On the other hand, according to Sinkovic et al. [15] purple seeds belong exclusively to the Andean germplasm, while pink, brown and black seeds are predominantly from the Mesoamerican germplasm. Cream, yellow and red seed colors are present in both gene pool groups. In this context, Chilean germplasm of runner bean could be composed of both Mesoamerican and Andean genetic pools. In general, most of the lines coming from the Center of Chile (lines 1, 4, 6, 7 and 8) and one from the Southern of the country (line 20) presented white flowers and white seeds, which indicates that they could correspond to the var. *albiflorus*, whereas those from Southern Chile (lines 19, 27 and 28) could correspond to the var. *coccineus*, given the red color of its flowers and purple seeds. The molecular mechanisms involved in the determination of seed color have been widely studied by bean breeders [32,33,34,35]. In common bean, the expression of seed color depends on the interaction between multiple genes and alleles with epistatic interactions, which define the different patterns and colors of the seeds within this species [32]. Among them, the dominant *P* (*Pigment*) gene and *ASPER* (Asp) genes are involved in the genetic control of seed color and shininess [35]. The recessive homozygous genotype (*pp*) generates individuals with flowers and seeds of white color [34]. This observation is in accordance with our results, given that the lines that showed white flowers presented white seeds too.

**Figure 3:**
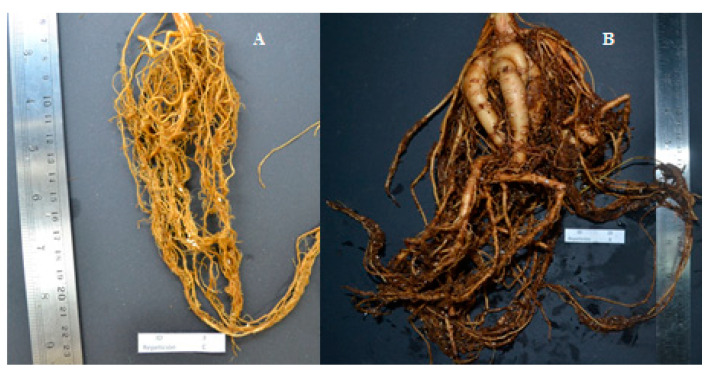
Root type present in *P. coccineus*. (**A**) A fibrous (fasciculated) root type belonging to line 3, and (**B**) a tuberous root type belonging to line 20.

The roots displayed brown, cream and white colors. All lines (except 3 and 4) presented nodules. It is possible that lines 3 and 4 were not inoculated with *rhizobia*, which allowed the identification of lines that are more prone to form nodules. Fifty percent of the lines had fibrous (fasciculated) roots (lines 1, 3, 4, 6 and 7), which could be classified as annual lines while the other 50% of the lines had tuberous roots (lines 8, 19, 20, 27 and 28). These lines can be multiplied vegetatively, and they have the potential to re-sprout the following year, being classified as perennial lines (Figure 3). Outside Central America and Mexico runner bean is cultivated as an annual crop, but in their natural habitats there are both wild and domesticated forms, and they are grown as a perennial crop in temperate humid and temperate semi-arid regions at altitudes higher than 1800 m.a.s.l. [5,36]. This could explain the difference between the two types of roots reported in this study, since Mexican cultivars can develop both types of roots, according to the area where they are grown, thus the Chilean germplasm could maintain phenotypic plasticity in root architecture [36]. In addition, it is known that runner bean roots are used as food in Central America, and in fact, the roots of *P. coccineus* var. *darwinianus* are eaten cooked [37]. Since *P. coccineus* var. *darwinianus* is distinguished by the tuberous root type [6], lines 8, 19, 20, 27 and 28 could correspond to that variety. However, soil characteristics such as structure, porosity and aeration, among others, can be very important in the final shape and size of the root system [38].

### 2.2. Quantitative Morphological Characterization

The multivariate analysis of ten morphological traits determined three principal components, which may explain 92.07% of the variability between the lines studied. Only the traits with values greater than 0.95 in the sum of the *r*^2^ obtained from the analysis of the principal components were selected (Table 3). The traits with the highest *r*^2^ values were: days to V2, number of seeds per pod, pod length, beak length, seed width and weight of 10 seeds, and a box plot is shown in the Figure S2. All the traits studied satisfied the ANOVA assumptions, excepting the length of beak and width of seed. Moreover, for the weight of the 10 seeds, not all the lines had enough repetitions, and therefore the trait was discarded for the ANOVA analysis. The estimates of degree of genetic determination (DGD) or broad heritability (*H*^2^) was higher than 60% for all the traits evaluated (except days to V2). The high determination makes these traits interesting for a runner bean breeding program. However, it is necessary to extend the study to a greater number of environments and repetitions of each line to obtain more precision on the genetic parameters evaluated.

Even though there is a close genetic relationship between the Chilean lines of *P. coccineus* evaluated, line 3 (from central Chile; INIA-La Platina) presents morphological differences compared to all the lines evaluated, which suggests a hybridization process and a possible heterosis effect that is independent of the genetic distance that exists between the parental lines [39]. In this context, line 3 showed an average of 4.9 seeds per pod, which was significantly (*p* > 0.05) higher than the rest of the lines evaluated, which varied between 1.7 (line 19) to 3.2 (line 1) seeds per pod. This result is in accordance with a previous study by Sinkovic et al. [15], who indicated that most of the accessions from South Eastern Europe had 2–3 seeds per pod. In addition, the average number of seeds per pod in a wild population of runner bean was 4.2 [40]. Therefore, the lines evaluated in this study exhibited intermediate values of seeds per pod, which may be due to various factors such as high air temperatures, self-incompatibility and the lack of pollinators, since it has been reported that the number of seeds per pod can increase up to 15% with the presence of pollinators [4,41]. Additionally, the number of seeds per pod is highly heritable in *Phaseolus* [42,43], which is in accordance with our result (*H*^2^ = 0.67). Therefore, this trait is a good selection criterion for improving seed yields of Chilean runner bean lines.

With respect to the shape of the pod, line 3 had the longest pod (16.5 cm), which was significantly higher than the other lines (10.3 to 13.5 cm). The pods of lines 19 and 20 are significantly shorter with pod lengths of 10.7 and 10.3 cm, respectively (Table 4). Our results are similar to those obtained in previous studies, as in Italian populations of runner bean that had pod lengths that averaged 13.8 cm [27]. Other studies indicate that the pod length varies between 10 and 15 cm in runner beans [5,15,44]. Among the three botanical varieties, var. *coccineus* is the one with the longest pods [5]. The lines evaluated in this study showed pod widths between 1.2 (line 6) and 1.9 cm (line 28), which is in agreement with previous reports where pod width varied between 1.5 and 2 cm [15,44]. Therefore, the shape of pods present in the Chilean lines is within the ranges expected for this species.

Regarding the length of the beak pod, the lines were grouped into two significantly different groups (*p* < 0.05). Lines 1, 3, 4, 6 and 7 had long beak pods whose values oscillated between 1.5 and 2.0 cm, while lines 8, 19, 20, 27 and 28 gave pods with short peaks, and their values varied between 0.7 and 0.52 cm (Table 4). The number of filled pods per plant is a very important yield component to increase bean yield. The lines evaluated had on average between 2.4 and 46 pods per plant for lines 28 and 4, respectively, suggesting that this trait generally has a low heritability in the population, and it is highly influenced by the environment. This is a similar result to that obtained by Kimani et al. [11], in which the number of pods per plant varied from 2 to 48 in advanced grain runner bean lines. Moreover, according to Bezerra et al. [45] the number of pods per plant decreases with increasing plant density, due to an intraspecific competition compromising photosynthesis.

One of the most important features of runner bean is the larger size of its seeds compared to common beans [4]. Traits related to the weight and shape of the seeds are very important in bean genetic improvement programs, since they provide the major determinants of the commercial acceptability of the varieties [15]. Among the traits related to seeds, the length varied between 1.89 (line 4) and 2.24 (lines 19 and 27) cm, while the seed width fluctuated between 1.05 (line 7) and 1.34 (line 27) cm. The average weight of 10 seeds was estimated at 8 and 15.5 g for the lines 4 and 19, respectively. These results are in agreement with previous reports for runner-bean accessions from Spain [8], Italy [16,27], Slovenia [44], Romania [46] and South Eastern Europe [15].

**Figure 4:**
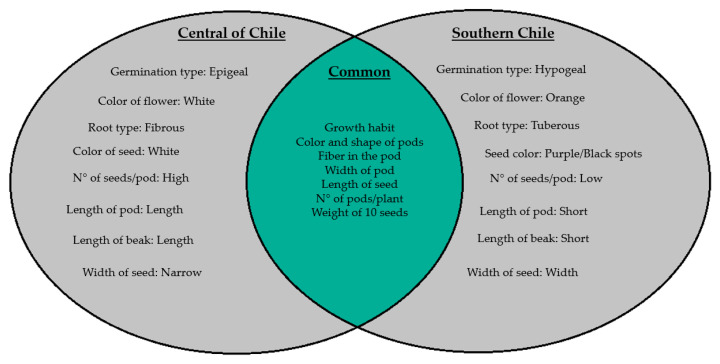
Summary of the similarities and differences between the *P. coccineus* lines evaluated for the most relevant morphological and agronomic traits. In the center are the similarities (common), while on the left and right the traits for the lines coming from the Center and South of Chile, respectively.

Finally, Figure 4 summarizes the similarities and differences between the *P. coccineus* lines evaluated for the most relevant morphological and agronomic traits.

### 2.3. Phenological Characterization

The morphological and physiological changes that a plant experiences during its biological cycle define the stages of development [40]. The analysis of the phenological development for the Chilean lines of runner bean evaluated in this study is shown in Table S1. For days to emergency, the earliest lines were 8, 20 and 28, which emerged before 13 days. Line 7 was the latest, which emerged after 23 days. The rest of the lines emerged between 14 and 22 days, not being significantly different between them. In runner bean, low temperatures delay the plant emergence, initiation of flowering and thus slow down the phenology [10]. For example, accessions from Spain, Portugal, Mexico and Rwanda were germinated under optimal (17 °C-day/15 °C-night) and sub-optimal (14 °C-day/8 °C-night) temperature conditions. On average, these accessions germinated 7.4 days after sowing under optimal conditions, while under suboptimal conditions the emergency was delayed to 28.8 days [7]. Therefore, it may be that the environmental conditions in which the lines were evaluated in this study were not optimal for this species. The earliness of a variety is given by the time necessary to reach a certain stage of development by the phenological stages achieved in a specific time. In this sense, line 28 is the earliest, with a total of 34 days until the appearance of the flower buds, while lines 7 and 27 are the latest, since they exceed 48 days. The rest of the lines did not differ significantly among them, and the emergence of the floral buds occurred between 43–45 days after sowing.

### 2.4. Molecular Characterization

The number of different alleles (*Na*), number of effective alleles (*Ne*), Shannon index (*I*), Nei’s gene diversity (*h*, [47]) and percentage of polymorphic loci (*P*) were estimated to assess genetic diversity levels in the lines of *Phaseolus* spp. evaluated (Table 5). The analyses were performed in two groups, one including only *P. coccineus* lines, and the other group including the *P. vulgaris* and the *P. lunatus* lines. In general, for the entire population, the ISSR markers were polymorphic and generated a total of 256 bands/alleles. In *P. coccineus*, 79.3% of the loci were polymorphic, the number of alleles per locus ranged from 20 to 26, and the size of the DNA fragments ranged from 200 to 3000 bp. The Nei’s gene diversity value was *h* = 0.237, and the Shannon index (*I*) was 0.367. In addition, similar levels of genetic diversity were identified between lines from Central and Southern Chile. In the group of *P. vulgaris* together with *P. lunatus*, 65.6% of the loci were polymorphic, and the *h* and *I* values were 0.214 and 0.327, respectively.

There is little information about the genetic diversity and structure of the wild and cultivated accessions of *P. coccineus* [3]. For example, Spataro et al. [1] estimated moderate levels of genetic diversity in Mesoamerican wild genotypes (*He* = 0.50) and landraces (*He* = 0.54) accessions using SSR markers. In contrast, Guerra-García et al. [2] estimated low levels of genetic diversity in the wild (*He* = 0.118–0.208), and cultivated (*He* = 0.134–0.199) Mexican populations using SNP markers. Similarly, low and moderate levels of genetic diversity have been encountered in European accessions of *P. coccineus* (*He* = 0.167–0.43; [1,5,16,17,18]). These findings confirm that selected *P. coccineus* accessions presented similar levels of genetic diversity in wild and cultivated populations, which has been little affected by the domestication process [2]. However, our results are affected by the size of the population, and the high genetic relationship that exists among the lines, since the lines of *P. coccineus* only have two centers of origin: Central Chile (INIA-La Platina) and Southern Chile (UACH and PUC-Villarrica).

**Figure 5:**
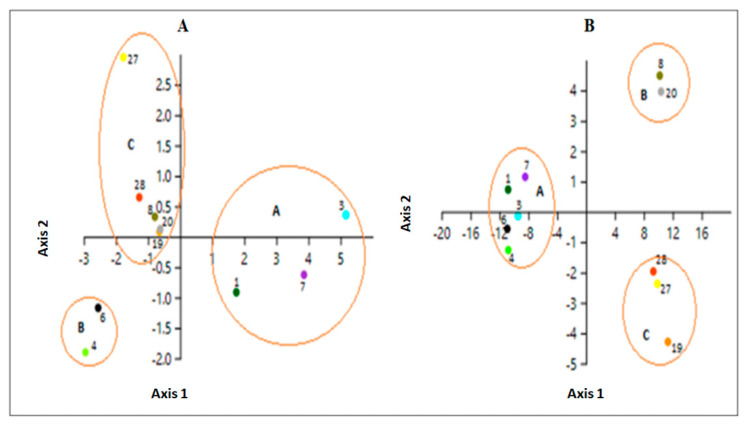
Graphs of the discriminant analysis using morphological (**A**) and molecular data (**B**). In both graphs, the *P. coccineus* lines were grouped into three groups (A, B and C). The lines are identified with their respective numbers.

**Figure 6:**
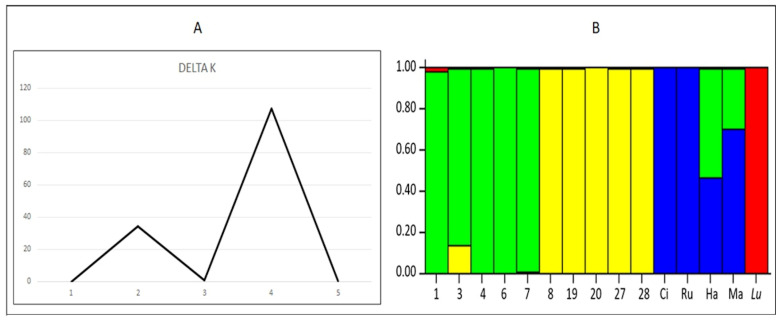
Bayesian clustering approach using all the evaluated genotypes (*P. coccineus*, *P. vulgaris* and *P. lunatus*). (**A**) result from the Evanno´s method, where *K* = 4 is the most likely number of genetic groups (largest delta *K*). (**B**) The estimated membership probability for each line to each different group is shown in the vertical axis. 1, 3, 4, 6, 7, 8, 19, 20, 27 and 28 are lines of *P. coccineus*; Ci: Cimarrón, Ru: Rubí; Ha: Hallado Alemán, and Ma: Mantenca are commercial varieties of *P. vulgaris*, and *Lu* is a line of *P. lunatus*.

According to the principal component and discriminant analysis, the lines evaluated were grouped into three groups using the morphological information. Lines 1, 3 and 7 belong to group A; lines 4 and 6 belong to group B; and lines 8, 19, 20, 27 and 28 belong to group C (Figure 5). Accordingly, the lines were differently grouped into three groups using the molecular information (Wilk´s Lambda = 0.001283), which may explain 63.48% of the variability. Lines 1, 3, 4, 6 and 7 were grouped in group A, lines 8 and 20 belonged to group C, and the lines 19, 27 and 28 at group B (Figure 5). Most of the lines belonging to group A (using morphological and molecular data) come from INIA-La Platina, and are characterized by having a greater number of pods per plant, although the size and weight of the seeds are lower compared to the other groups. Lines of the groups B and C come from the Southern Chile (UACH and PUC-Villarica), and they are characterized by fewer pods per plant and seeds per pod, but the size and the weight of their seeds are bigger than group A. Finally, using the complete set of genotypes (15 lines), the most probable number of genetically differentiated groups (*K*) was 4 (Figure 6A). The lines of *P. coccineus* were classified into two different groups according to their place of origin (Central and Southern Chile). Although line 8 belongs to Central Chile (INIA-La Platina), it has agronomic traits similar to the lines from Southern Chile, indicating its possible genetic origin. The *P. vulgaris* varieties were all grouped in the same group, while the *P. lunatus* line was classified in a different group (Figure 6B).

In conclusion, although the findings of this study should be taken with caution due to the low number of lines and replicates evaluated, this is one of the first works which generate relevant and novel information on the morphological, agronomic and genetic characterization of the *P. coccineus* germplasm present in Chile. Line 3 (from Central of Chile) presents some morphological (qualitative and quantitative) traits different from the rest of the lines evaluated, suggesting a hybridization process and with a possible heterosis effect. Finally, it was determined that the evaluated *P. coccineus* lines show moderate morphological, agronomic and genetic variability, which allows this specie to be a candidate for genetic improvement programs due to its culinary properties, or to improve some traits in other *Phaseolus* species.

## 3. Materials and Methods

### 3.1. Plant Material

A total of ten lines of runner bean (*Phaseolus coccineus* L.) from different research centers (Central and Southern Chile) were obtained and available for the development of this work (Table S2). Six lines (1, 3, 4, 6, 7 and 8) were obtained from the Instituto de Investigaciones Agropecuarias (Central of Chile; INIA-La Platina), the lines 19 and 20 were provided by Universidad Austral de Chile (Southern Chile; UACH), and two lines (27 and 28) were provided by the Pontificia Universidad Católica de Chile (Southern Chile; PUC-Villarica). In addition, a line of *P. lunatus* (LU), and four cultivars of *P. vulgaris* (Cimarrón, Rubí, Hallado Alemán and Manteca) collected from the Navidad County, located in the O’Higgins Region in Chile, were included.

### 3.2. Experiment Design and Agronomic Management

The runner bean lines were sown in a greenhouse belonging to the Faculty of Agronomy and Forest Engineering (PUC-Santiago) on 3 June 3 2015, and 60-day-old seedlings were transplanted into 25-liter pots using as substrate a mixture of perlite and peat (Kekkilä DSM 0; pH = 5.5; see description http://protekta.cl/img/cms/PDF/DSM%200.pdf, accessed 2 June 2021) in a ratio of 1:2. The experimental design was a randomized complete block design with six replications. The irrigation was applied according to stages of development and growth of each line. The fertilization was carried out at the time of transplanting with 200 kg N/ha, 100 kg of P_2_O_5_/ha and 60 kg of K_2_O/ha [48]. The presence of thrips (*Thrips* spp.) and whiteflies (*Bemisia* spp.) was controlled in a traditional way with insecticide applications in the greenhouse. Lines 1, 3, 4, 6 and 7 were harvested on 5 January 2016, while lines 8, 19, 20, 27 and 28 were harvested on 20 April 2016.

### 3.3. Morphological and Molecular Characterization

Morphological traits were analyzed according to “TG/9/5” guidelines of the International Union for the Protection of New Varieties of Plants (UPOV, 2003; https://www.upov.int/edocs/tgdocs/es/tg009.pdf, accessed 15 June 2021), using the descriptors least affected by the environment. The phenological traits evaluated were days to emergence, germination type, two-leaf stage (V2), four-leaf stage (V4), flower bud, flower color, height of branches and type of growth; for traits related to pods, the length and width of the pod, number of pods per plant, length of pod peak, number of seeds per pod and color and shape of the pod were evaluated; for traits related to seeds, the weight of 10 random fresh seeds per plant, as well as color, width, length, shape and color of the hilum were evaluated; and regarding the roots, the color, type and presence or absence of nodules were evaluated. All traits were evaluated based on the six replicates per line.

Genomic DNA was extracted from young leaves of one individual from each line. The individuals were selected according to those that met the highest number of morphological traits evaluated and were representative for each line. DNA was extracted using a modified CTAB (trimethyl ammonium cetyl (hexadecyl) bromide) method [49], and quantified by fluorometry (Qubit^®^ 3.0, Thermo Fisher Scientific Inc., Waltham, MA, USA). Total DNA was diluted to 70 ng/mL using ultra-pure water (GIBCO^®^, Invitrogen, Carlsbad, CA, USA) for each sample, which were stored at −20 °C until further analysis. The integrity of the extracted DNA was checked by 2% agarose gel electrophoresis in TAE 1X buffer (Buffer TAE 1X, 90 V), stained with Gel Red™ and exposed to UV light to visualize genomic DNA.

A total of 12 ISSR markers [50] from the UBC Primer Set #9 (Microsatellite) designed by the University of British Columbia (UBC) in Canada were evaluated. These markers showed a high number of polymorphic bands/alleles (Table S2). The PCR amplification was carried out in 25 μL final reaction volume containing 100 ng of template DNA, 1X PCR buffer with 2.0 mM MgSO_4_, 0.75U Taq DNA polymerase, 0.2 mM dNTPs, and 0.47 µM of each primer. The PCR amplification was performed using a VeritiTM Thermal Cycler (Applied Biosystems, CA, USA), and utilizing the following program: hold at 95 °C for 1 min, and then 35 cycles of PCR at 95 ℃ for 20 s, annealing (Tm) at an appropriate temperature for each marker for 30 s, and 68 °C for 5 min, followed by a final extension for 15 min at 72 ℃. Finally, the PCR products were analyzed by gel electrophoresis in 2% agarose (Buffer TAE1X, 90 V), stained with Gel Red™ and exposed to UV light to visualize PCR products. The 100 bp molecular weight marker (Thermo Fisher Scientific Inc., Waltham, MA, USA) was used to estimate the molecular weight of the DNA fragments (Figure S3). Finally, the ISSR bands were treated as an independent locus and a binary matrix was obtained by visual scoring of the ISSR markers based on the presence (1) or absence (0) of a particular band.

### 3.4. Statistical Analysis

For the morphological and phenological analyses, the traits were grouped according to whether they were qualitative or quantitative. The Shapiro–Wilk (normality) and Levene (homoscedasticity) tests were performed to evaluate the analysis of variance (ANOVA) assumptions. A principal component analysis (PCA) was performed using the Past3 program [51] to determine the presence of groups between the lines studied. In addition, a discriminant analysis was carried out to verify the proposed grouping. Finally, Tukey’s test (*p* > 0.05) was performed in the InfoStat program [52] to evaluate significant differences among the traits that were significant (from the correlation matrix) in PCA. The analysis of the qualitative traits was conducted through the frequency that they showed in the six replicates of the different lines.

For molecular analysis, the presence or absence of a given band was scored as 1 and 0, respectively (Supplementary File 1). The genetic distances among the individuals were calculated using GenAlEx 6.5 software [53], which were used to perform a principal coordinate analysis (PCoA). The three coordinates that explained the highest percentage of variability between loci were selected, and the analysis was carried out using Past3 software [51]. The significance of the discriminate analysis was determined by Wilks lambda with the NCSS program (NCSS Kaysville, UT). The genetic diversity of the Chilean *Phaseolus* spp. lines evaluated was estimated through the number of different alleles (*Na*), number of effective alleles (*Ne*), Shannon index (*I*), Nei’s gene diversity (*h*) and percentage of polymorphic loci (*P*) using the POPGENE 32 program [54]. Nei’s genetic diversity is defined as

(1) $$h=1-\sum p_{i}^{2}\;$$

where (in the case of dominant molecular markers)$\;p_{i}$ is the population frequency of each allele (1 and 0) at locus *i* [55].

Nei’s genetic diversity is a simple measure of genetic variability, and is used when the samples are small [55]. A preliminary analysis on the genetic structure of the lines evaluated was carried out in STRUCTURE 2.3.4 [56]. The Bayesian model-based clustering approach was performed using all the evaluated genotypes (*P. coccineus*, *P. vulgaris* and *P. lunatus*) to infer the most probable number of genetic groups. The number of subpopulations (*K*) was set from 1 to 5 based on admixture, and correlated allele frequencies models, and ten runs per *K* were conducted separately. Each run was performed with 1,000,000 Monte Carlo Markov Chain (MCMC) replicates and a burn-in period of 10,000 iterations. A step of 100 iterations was used to avoid autocorrelation. The true *K* value was inferred using the method proposed by Evanno et al. [57], and it was plotted in Microsoft Excel^®^.

## Figures and Tables

**Table 1:** Qualitative external traits evaluated in the different lines of *P. coccineus* based on the type of germination, flower color, type of branching and growth habit.

Lines	Germination Type	Color of Flower	Branch Type	Growth Habit
1	Hypogeal	White	Evenly distributed (upper and lower)	Indeterminate
3	Epigeal	Purple	Evenly distributed/lower *	Indeterminate/determinate *
4	Epigeal	White	Evenly distributed	Indeterminate
6	Epigeal	White	Evenly distributed	Indeterminate
7	Epigeal	White	Evenly distributed	Indeterminate
8	Hypogeal	White	Evenly distributed	Indeterminate
19	Hypogeal	Orange	Evenly distributed	Indeterminate
20	Hypogeal	White	Evenly distributed	Indeterminate
27	Hypogeal	Orange/White	Evenly distributed/upper *	Indeterminate
28	Hypogeal	Orange/White	Evenly distributed/upper *	Indeterminate

* Lines that presented the same frequency for two classifications within the traits studied.

**Table 2:** Qualitative traits related to the pod, seed and root of the *P. coccineus* lines. The traits that are presented in the table are those that were found more frequently within each line (from six plants each). The traits related to the root were evaluated at the time of harvest.

	Pod-Related Traits			Seed-Related Traits				Root-Related Traits		
Lines	Color	Shape	Fibers	Color	Longitudinal Cut	Cross-Section Cut	Hilum Color	Color	Nodules	Type
1	Beige	Concave	Yes	White	Kidney	Narrow elliptical	White	Brown	Yes	Fibrous
3	Beige/Purple spots	Concave	Yes	Cream/Black spots	Kidney	Narrow elliptical	Cream	Brown	No	Fibrous
4	Cream/Brown spots	Concave	Yes	White	Kidney	Narrow elliptical	White	Brown	No	Fibrous
6	Cream	Concave	Yes	White	Elliptical Circular	Narrow elliptical	White	Brown	Yes	Fibrous
7	Beige	Concave	Yes	White	Elliptical Circular	Narrow elliptical	White	Brown	Yes	Fibrous
8	Beige	Concave	Yes	White	Kidney	Elliptical	White	Cream	Yes	Tuberous
19	Beige	Concave	Yes	Purple/Black spots	Kidney	Circular	Cream	Cream	Yes	Tuberous
20	Beige	Concave	Yes	White	Rectangular	Elliptical	White	White	Yes	Tuberous
27	Beige	Concave	Yes	Purple/Black spots	Kidney	Elliptical	White	Cream	Yes	Tuberous
28	Beige	Concave	Yes	Purple/Black spots	Kidney	Elliptical	Cream	Cream	Yes	Tuberous

**Table 3:** Values obtained from the ANOVA analysis performed in Past3 for each of the variables that had *r*^2^ values greater than 0.95.

Trait	Sum of Squares	d.f.	Mean Squares	*p* Value	GDG
Days to V2	1429.280	9	158.809	0.007	55.85%
N° of seeds per pod	42.600	9	8.088	0.001	67.83%
Pod length	142.424	9	7.162	0.007	64.46%
Beak length	18.674	9	2.074	3.69 × 10^−16^	95.04%
Seed width	0.535	9	0.059	0.004	72.15%

V2—primary leaves; GDG—Degree of genetic determination or heritability in the broad sense (*H*^2^) expressed as a percentage; d.f.—Degrees of freedom.

**Table 4:** Quantitative traits related to the size and number of pods and seeds of *P. coccineus* lines. The values are the averages of the six repetitions for each line. The letters next to the means indicate the significance for the difference between the means of each trait analyzed using the Tuckey test. Only different letters mean significant differences. Traits without letters do not vary significantly between genotypes.

	Pod-Related Traits					Seed-Related Traits		
Lines	N° of Seeds/Pod	Length (cm)	Length of Beak (cm)	Width (cm)	N° of Pods/Plant	Length (cm)	Width (cm)	Weight of 10 Seeds
1	3.26 b	13.46 ab	1.76 ab	1.5	35.67	1.95	1.08 bc	8.5
3	4.92 a	16.48 a	1.49 b	1.66	16.17	1.94	1.07 bc	8.68
4	2.87 b	11.76 bc	2.06 a	1.45	46.17	1.89	1.06 c	8.07
6	2.78 b	12.20 bc	1.86 ab	1.28	26.83	1.91	1.07 bc	8.88
7	3.13 b	12.59 bc	1.70 b	1.39	26.57	1.92	1.05 c	8.54
8	2.19 b	10.47 bc	0.58 c	1.53	3	2.06	1.23 abc	13.55
19	1.77 b	10.75 c	0.52 c	1.82	6	2.24	1.29 a	15.46
20	2.02 b	10.34 c	0.59 c	1.78	4	1.98	1.25 ab	11.65
27	1.85 b	11.84 bc	0.69 c	1.77	8.71	2.24	1.34 a	14.4
28	2.50 b	12.87 bc	0.70 c	1.87	2.4	2.07	1.23 abc	11.89

**Table 5:** Genetic diversity indexes of the different lines of *Phaseolus* evaluated in this study.

Species	Region	N	*Na*	*Ne*	*I*	*h*	*P*
*P. coccineus*	Central of Chile	6	1.652 ± 0.477	1.399 ± 0.334	0.319 ± 0.262	0.208 ± 0.182	65.23%
	Southern of Chile	4	1.582 ± 0.494	1.366 ± 0.361	0.322 ± 0.286	0.216 ± 0.198	58.20%
*P. coccineus*	Overall	10	1.793 ± 0.406	1.378 ± 0.314	0.367 ± 0.237	0.237 ± 0.169	79.30%
*P. vulgaris + P. lunatus*		5	1.656 ± 0.475	1.357 ± 0.358	0.327 ± 0.266	0.214 ± 0.187	65.62%

***N***—number of individuals; ***Na***—Number of different alleles; ***Ne***—Number of effective alleles; ***I***—Shannon index; ***h***—Nei’s gene diversity; ***P***—Percentage of polymorphic loci.

## Data Availability

Not applicable.

## Supplementary Materials

The following are available online at https://www.mdpi.com/article/10.3390/plants10081688/s1, Figure S1: Shapes and colors of the pods in the *P. coccineus* lines evaluated, Figure S2: Boxplot for the traits with values greater than 0.95 in the sum of the *r*^2^ obtained from the analysis of the principal components, Figure S3: Agarose gel showing the amplification pattern of the UBC-835 primer, Table S1: Analysis of the phenological states of the *P. coccineus* lines, Table S2: Accession names and origins of the *Phaseolus* spp. lines evaluated in this study, Table S3: List of molecular markers (ISSR) used to genotype the *Phaseolus* spp. lines evaluated in this study.
