# Supplementary material for: Morphological and Molecular Characterization of Selected Chilean Runner Bean (Phaseolus coccineus L.) Genotypes Shows Moderate Agronomic and Genetic Variability
Source: Plants (Basel). 2021 Aug 17;10(8):1688. doi: 10.3390/plants10081688 (PMC8400864; doi:10.3390/plants10081688)
Supplement: Supplementary file 1 [file plants-10-01688-s001.zip › plants-1331347-supplementary.pdf]

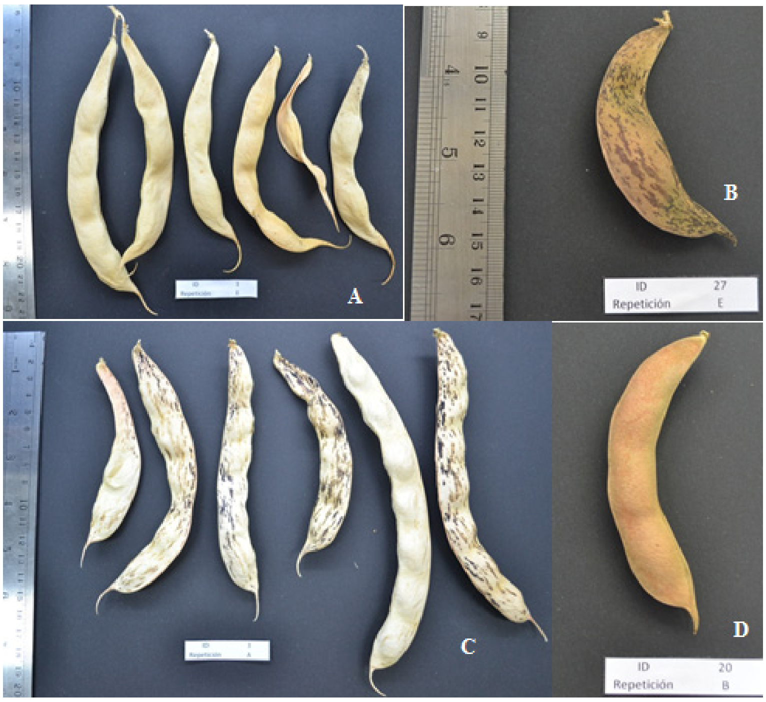

Figure S1. Shapes and colors of the pods in the *P. coccineus* lines evaluated. (**A)** Cream pods on line 1. **B)** Concave pods with purple spots present on line 27. **C)** Pods with different pattern and spot distribution on line 3. **D)** Beige pods without spots present on multiple lines, represented by line 20.

Lines	Emergency (V1)	V2	V4	Flower bud	Vegetative period	Total cultivation period
1	21,5 AB	38,33 AB	54,17 A	45,4 AB	54,17 A	216
3	19,4 ABC	40,33 A	52,67 AB	46 AB	52,67 A	216
4	16,83 ABC	33,67 ABC	51,33 ACB	45,83 AB	51,33 A	216
6	14,8 ABC	33 ABC	47,8 ABCD	43,5 AB	47,8 AB	216
7	22,33 A	41,8 A	48 ABC	50,6 A	50,6 AB	216
8	12,17 C	29,6 BC	41,83 CD	42,2 AB	42,2 AB	323
19	13 BC	29,4 BC	41,67 BCD	44,5 AB	44,5 AB	323
20	13 C	28,33 BC	42,83 CD	45,67 AB	45,67 AB	323
27	16,83 ABC	30,83 ABC	44,33 ABCD	48,83 A	48,83 AB	323
28	11,67 C	26,33 C	37,33 D	34,33 B	37,33 B	323

Table S1. Analysis of the phenological states of the *P. coccineus* lines. It shows the days after sowing in which the different states are reached by the different lines. The letters indicate significant differences between the lines by Tuckey's test.

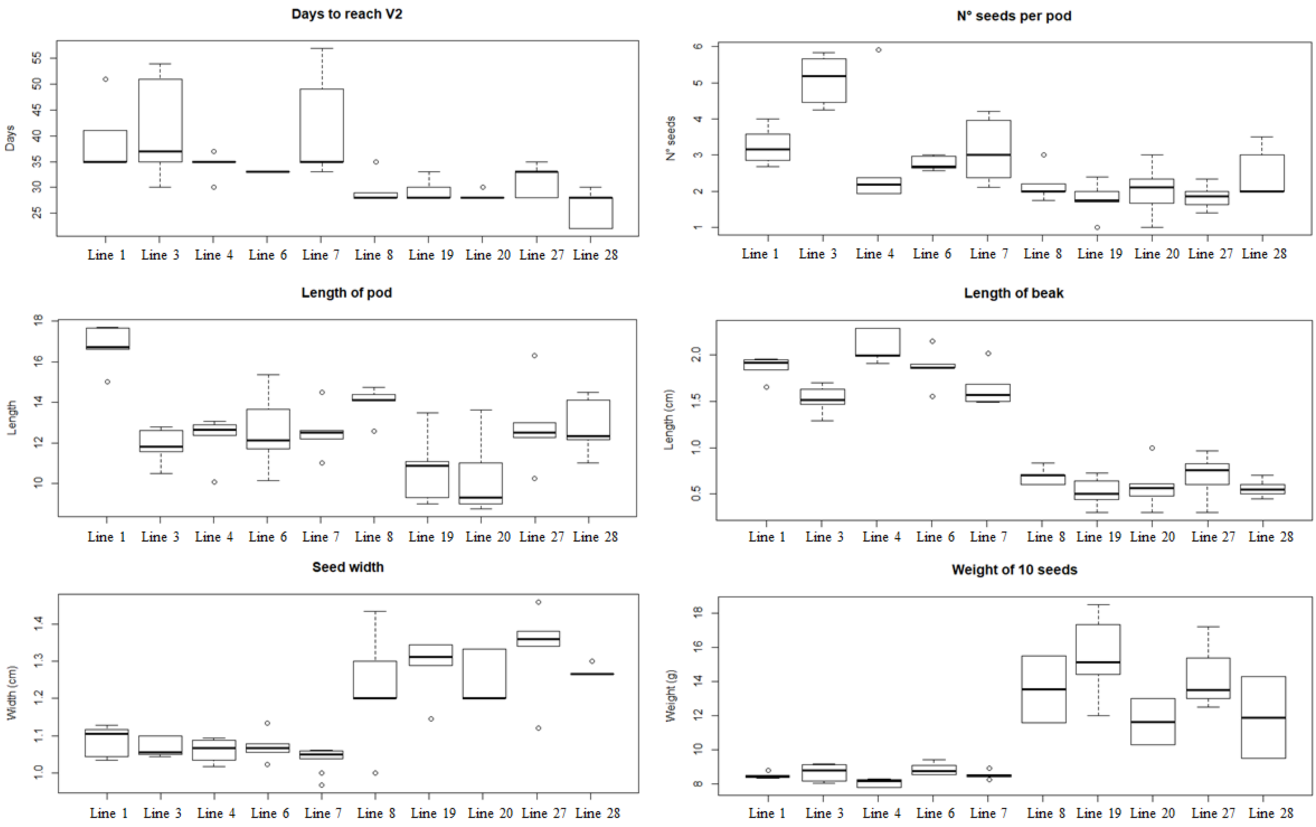

Figure S2. Boxplot for the traits with values greater than 0.95 in the sum of the r^2 obtained from the analysis of the principal components. The traits were days to V2, number of seeds per pod, pod length, beak length, seed width and weight of 10 seeds.

Table S2. Accession names and origins of the *Phaseolus* spp. lines evaluated in this study. The lines 1, 3, 4, 6, 7, 8, 19, 20, 27 and 28 correspond to *P. coccineus* (*Pc*), the *LU* line corresponds to *P. lunatus* (*Pl*) and the Cimarrón, Rubí, Hallado Alemán and Manteca cultivars correspond to *P. vulgaris* (*Pv*).

Lines	Accession name	Origin	coordinates	*Phaseolus* spp
1	RG- 29	INIA Platina	33°34'23"S; 70°37'15"W	*Pc*
3	RG- 124	INIA Platina	33°34'23"S; 70°37'15"W	*Pc*
4	RG- 176	INIA Platina	33°34'23"S; 70°37'15"W	*Pc*
6	RG- 596	INIA Platina	33°34'23"S; 70°37'15"W	*Pc*
7	RG- 620	INIA Platina	33°34'23"S; 70°37'15"W	*Pc*
8	RG- 90035	INIA Platina	33°34'23"S; 70°37'15"W	*Pc*
19	4292CON90PHA1187	UACH	39°48'26"S; 73°15'04"W	*Pc*
20	4328CON90PHA1223	UACH	39°48'26"S; 73°15'04"W	*Pc*
27	Villarica6	PUC Villarica	39°27'74"S; 72°22'53"W	*Pc*

28	Villarica7	PUC Villarica	39°27'74"S;<br>72°22'53"W	*Pc*
LU	Navidad	Navidad	33°56'00"S;<br>71°50'00"W	*Pl*
Cimarrón	Commercial	Commercial		*Pv*
Rubí	Commercial	Commercial		*Pv*
Hallado Alemán	Commercial	Commercial		*Pv*
Manteca	Commercial	Commercial		*Pv*

Table S3. List of molecular markers (ISSR) used to genotype the *Phaseolus* spp. lines evaluated in this study.

Primer/marker	UBC Primer	Sequence	Tm (°C)
P1	808	AGAGAGAGAGAGAGAGC	50
P3	812	GAGAGAGAGAGAGAGAA	50
P5	817	CACACAACACACACACAA	50
P6	823	ACACACACACACACACT	48
P8	834	AGAGAGAGAGAGAGAYT	52
P9	835	AGAGAGAGAGAGAGAGYC	54
P10	842	GAGAGAGAGAGAGAGYG	54
P11	844	CTCTCTCTCTCTCTCTRC	54
P12	846	CACACACACACACACART	50
P13	850	GTGTGTGTGTGTGTGTYC	52
P16	881	GGGTGGGGTGGGGTG	50
P18	887	DVDTCTCTCTCTCTCTC	50

UBC primer: identifier number of the University of British Columbia (UBC) primer Set # 9. Tm: annealing temperature for each marker

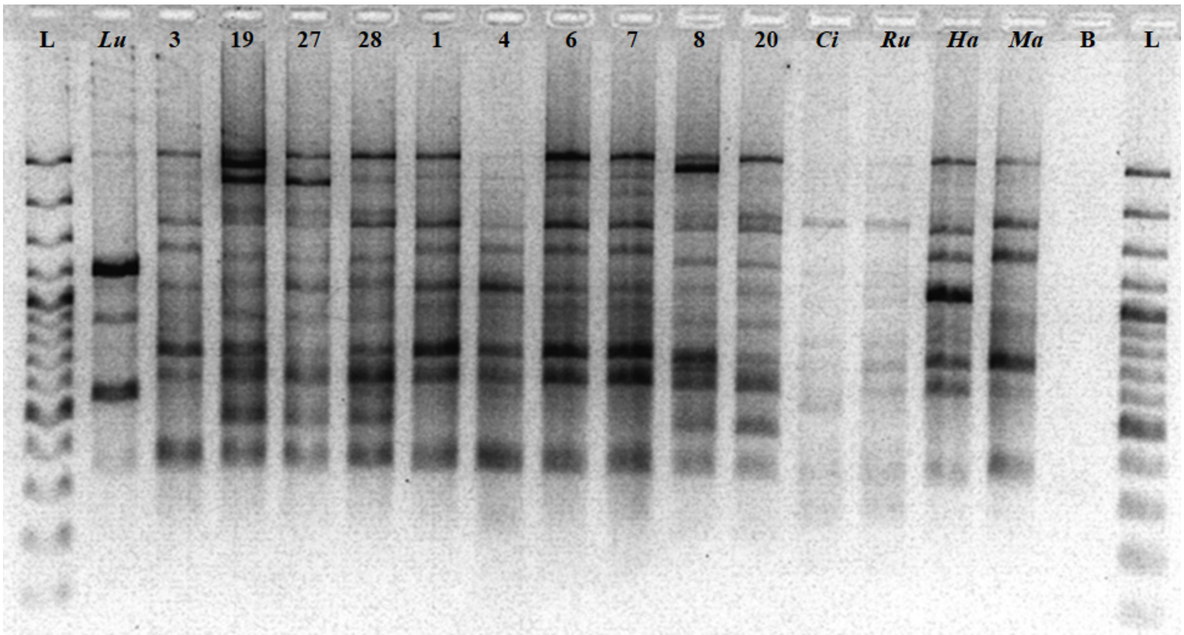

Figure S3. Agarose gel showing the amplification pattern of the UBC-835 primer. **L**: Ladder 100 bp (6X Orange DNA Loading Dye, Thermo Fisher Scientific Inc); ***Lu***: *P. lunatus*; Lanes 3-12: corresponds to the 10 lines of *P. coccineus* evaluated. Lanes 13-16: varieties of *P. vulgaris* evaluated (***Ci***: Cimarrón; ***Ru***: Rubí; ***Ha***: Hallado Alemán, and ***Ma***: Manteca). **B**: Blank.
